# A Coupled Modeling Approach for Water Management in a River–Reservoir System

**DOI:** 10.3390/ijerph16162949

**Published:** 2019-08-16

**Authors:** Zhenyu Zhang, Jinliang Huang, Min Zhou, Yaling Huang, Yimin Lu

**Affiliations:** 1 Coastal and Ocean Management Institute, Xiamen University, Xiamen 361102, China; 2 Spatial Information Research Center of Fujian Province, Fuzhou University, Fuzhou 350108, China

**Keywords:** Hydrological Simulation Program Fortran (HSPF), Environmental Fluid Dynamics Code (EFDC), modeling, river-reservoir continuum, watershed management

## Abstract

A coupled model is an effective tool to understand the nutrient fate associated with hydrodynamic and ecosystem processes and thereby developing a water resource management strategy. This paper presents a coupled modeling approach that consists of a watershed model and a hydrodynamic model to evaluate the nutrient fate in a river–reservoir system. The results obtained from the model showed a good agreement with field observations. The results revealed that the Shuikou reservoir (Fuzhou, China)exhibited complicated hydrodynamic characteristics, which may induce the pattern of nutrient export. Reservoirs can greatly lower water quality as a result of decreasing water movement. Three scenarios were analyzed for water management. The NH_3_-N (Ammonia Nitrogen) decreased sharply in the outlet of Shuikou reservoir after NH_3_-N level in its tributary was reduced. After removing the farming cages, the water quality of the outlet of Shuikou reservoir was improved significantly. The DO (Dissolved Oxygen) had increased by 3%–10%, NH_3_-N had reduced by 5%–17%, and TP (Total Phosphorus) had reduced by 6%–21%. This study demonstrates that the proposed coupled modeling approach can effectively characterize waterway risks for water management in such a river–reservoir system.

## 1. Introduction

Water management is an important issue in public policy, and many efforts have been made to ensure security of water supply [1,2,3,4,5]. Numerical models have been proved to be effective tools that can characterize waterway risks at different temporal–spatial scales and may provide better information for water management [6,7]. They are frequently used in fluvial research/engineering and can be viable to simulate water quality and pollutant transport in water environments [8,9]. The realistic simulation of pollution accidents can provide the decision-makers with helpful information for a timely response to emergencies.

A number of watershed and receiving water quality models have been developed since 1925 when Streeter and Phelps built the first water quality model to manage river pollution [10]. In recent years, many complicated water quality models have been used to track pollutant transport in the watershed, such as the Water Quality Assessment Simulation Program (WASP) [11,12], the Environmental Fluid Dynamics Code (EFDC) [13,14,15], the Soil and Water Assessment Tool (SWAT) [16,17,18], and the Hydrological Simulation Program Fortran (HSPF) [19,20]. In certain cases, a single model cannot fully delineate the complicated eco-hydrologic and biogeochemical processes within a watershed spatially composed of river, creek, reservoir, etc. Therefore, many modelers have realized that a coupled modeling approach in aquatic environment can improve the simulation of hydrodynamics and movement of pollutants [12,21,22].

China has been facing serious challenges in the conflict between irrigation water demand and spatiotemporally heterogeneous precipitation [23]. In the past few decades, many reservoirs and hydropower stations have been built to solve these problems. The Shuikou hydropower station (Fuzhou, China), the largest hydropower station in the Eastern China, has modified the stream flow regime and changed the riverine system of the Minjiang River watershed (Fujian, China). In addition, intensified human activities, such as cage farming and damming along the tributaries of Minjiang River watershed, have accelerated the degradation of water quality in the watershed (Figure S1). Therefore, an integrated management method is in high demand for identifying the potential pollution sources that impact water quality in such a system [4,5,24]. The objective of this study was to present a coupled modeling approach in a river–reservoir continuum. In addition, this study could also demonstrate the usefulness of the proposed approach in watershed management.

## 2. Materials and Methods

### 2.1. Study Area

**Figure 1:**
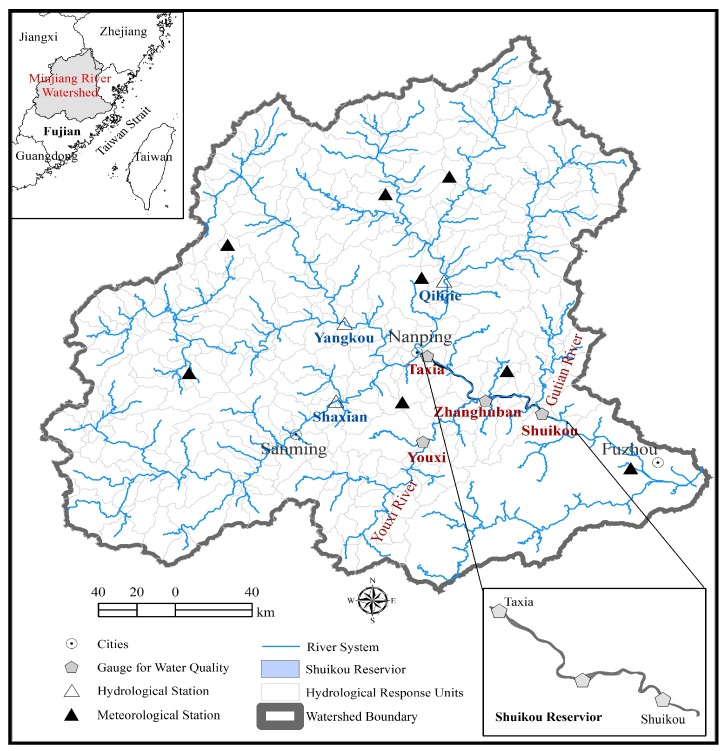
Study area.

Minjiang River Watershed (Figure 1) was selected as the test site. It covers 60,992 km^2^ and is located in a subtropical zone with a monsoon climate. The annual average temperature is 16–20 °C and annual average precipitation is 1617 mm, of which 70% occurs between April and September. The average annual runoff of the Minjiang River Watershed is 1980 m^3^/s. As the source of water of residential, industrial and agricultural activities, it supplies more than 12 million residents which accounts for approximately half of the population in Fujian Province [7]. The Shuikou hydropower station, was built along the mainstream of the Minjiang River. The dam of Shuikou hydropower station modified the streamflow regime of the Minjiang River in 1993 and subsequently formed a huge artificial lake (i.e., the Shuikou reservoir). The newly formed artificial lake is good for cage farming which has caused continuing deterioration of water quality in the last several decades and has posed a serious threat to local water security in the Minjiang River watershed. Youxi Creek, one of the tributaries of Minjiang River, was covered by the 3816 cages within more than 18,000 m^2^. In order to inhibit the deterioration of water quality, the local government has attempted to remove the farming cages in the Shuikou reservoir since 2018. Based on the survey of the last decade, the Shuikou reservoir overall has good water quality and more than 58% of the samples were labeled as Class III (Figure S2).

### 2.2. Model Description

A coupled modeling approach that consists of a watershed model (i.e., Hydrological Simulation Program Fortran (HSPF)) [25] and a hydrodynamic model (i.e., Environmental Fluid Dynamic Code (EFDC)) [8,9] was developed in this study in a typical river–reservoir system.

#### 2.2.1. Watershed Model—HSPF

**Figure 2:**
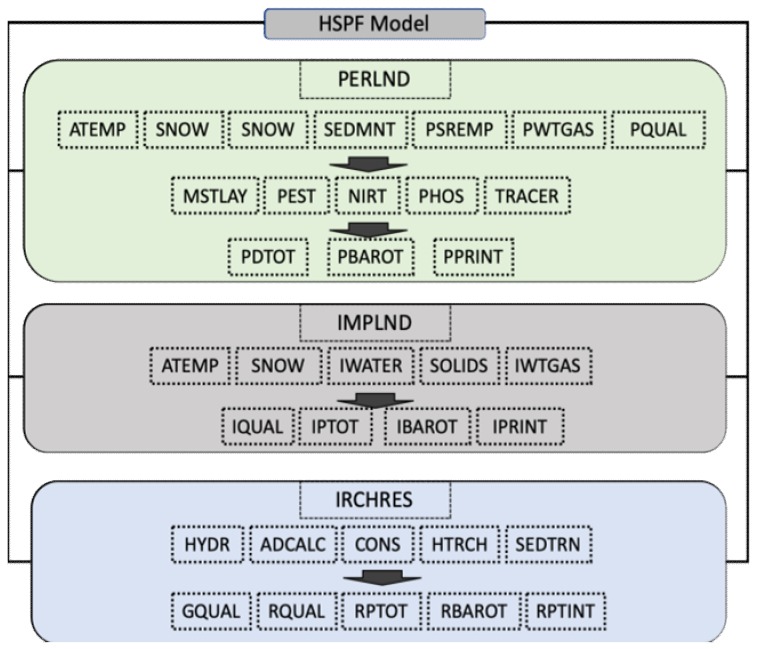
Structure chart for the Hydrological Simulation Program Fortran (HSPF) model (modified from Bicknell et al. [26]).

HSPF was employed to simulate streamflow and water quality at a watershed scale. It can provide boundary conditions to simulate the water and pollutants movement in the riverine system. HSPF is a set of computer codes developed by the US EPA (United States Environmental Protection Agency) which is based on the Stanford Watershed Model IV. HSPF is a semi-distributed, deterministic, continuous and physically-based model. The PERLND (Permeable Land Segments), IMPLND (Impermeable Land Segments) and RCHRES (Free-flow Reaches) modules are three main modules of HSPF to simulate permeable land segments, impermeable land segments and free-flow reaches, respectively (Figure 2) [26]. This model is useful in the evaluation of the hydrological process and nutrient transport in watersheds.

#### 2.2.2. EFDC Model in Riverine System

The EFDC is composed of a hydrodynamic module and a water quality module. It was used to simulate hydrodynamic and pollutant transport in a riverine system. The model can reflect temporal and spatial variation of water quality in the whole riverine system. EFDC can solve the depth average, free-surface, Reynolds-averaged Navier–Stokes equation using a second order accurate finite difference scheme on a staggered grid. This model works with orthogonal curvilinear coordinates in the vertical direction and the governing mass-balance equation is based on the conservation of mass. This equation can be expressed as follows [27].

(1) $$\partial_{t}C+\partial_{x}(\mu C)+\partial_{y}(\vartheta C)+\partial_{z}(\omega C)=\partial_{x}(K_{x}\partial_{x}C)+\partial_{y}(K_{y}\partial_{y}C)+\partial_{z}(K_{z}\partial_{z}C)+S_{C}$$

where *C* is the concentration of the water quality state variable; μ, ϑ and ω are the velocity components in the x, y and z directions, respectively; K_x_, K_y_ and K_z_ are the turbulent diffusivities in the x, y and z directions, respectively; and S_c_ is the internal and external sources and sinks per unit volume.

### 2.3. Model Configuration

**Figure 3:**
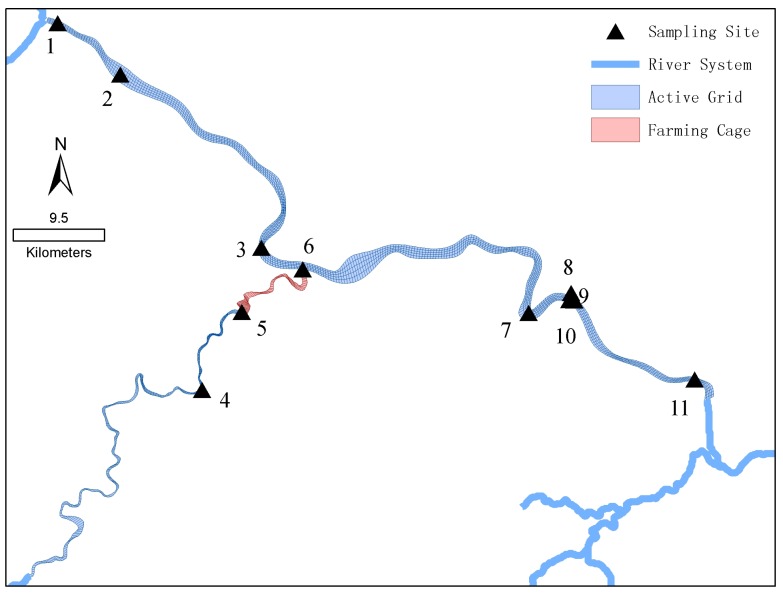
Model grids of the Environmental Fluid Dynamics Code (EFDC).

The watershed was discretized into 319 sub-basins (hydrological response units) with dominant land use and soil classification (Figure 1, Figures S3 and S4). Orthogonal grids were used to represent the geometry of the Shuikou reservoir (Figure 3). The EFDC model was set by allocating 2250 active grids (Figure 3). The runoff of the Minjiang River watershed was simulated by the HSPF model which was used as one of the initial conditions for the EFDC. In this study the output of the HSPF in the Taxia (Nanping, China), Youxi (Sanming, China) and Gutian Rivers (Ningde, China) (shown in Figure 1) was set as the initial boundary of the EFDC model.

### 2.4. Model Calibration and Validation

The models used in this study were calibrated and verified through matching the simulation results with the filed measured data during the periods of 2005–2008 and 2009–2012. Meteorological data including (precipitation, evaporation, temperature, wind speed, cloud cover) were obtained from the 21 weather stations in the Minjiang River watershed over the period 2005–2012 (Figure 1 and Figure S5). Three hydrologic stations of the Minjiang River watershed were selected to calibrate and validate the HSPF model (Figure 1). The Nash–Sutcliffe coefficient of efficiency (NSE) and coefficient of determination were used to evaluate the performance of the HSPF model.

(2) $$\mathrm{NSE}=1-\frac{\sum_{i=1}^{n}{\left(Q_{obs}-Q_{cal}\right)}^{2}}{\sum_{i=1}^{n}{\left(Q_{obs}-Q_{ave}\right)}^{2}}$$

where *Q_obs_*, *Q_cal_*, *Q_ave_* and *n* are the measured data, simulated data, average data and the number of the data respectively.

The relative error (RE) was employed to evaluate the performance of the HSPF model in terms of the water quality. The calibration period and validation period of the water quality of the HSPF model was 2005–2008 and 2009–2012 respectively.

(3) $$\mathrm{RE}=\frac{1}{n}\sum_{i=1}^{n}\left|Q_{obs}-Q_{cal}\right|$$

where *Q_obs_*, *Q_cal_* and *n* is the measured data, simulated data, average data and the number of the data respectively.

For modeling the riverine system, four water quality stations (namely, Taxia, Youxi, Zhanghuban and Shuikou) were further selected to calibrate and validate the water quality module of the EFDC model (Figure 1). Three water quality variables (i.e., DO (Dissolved Oxygen), NH_3_-N (Ammonia Nitrogen) and TP(Total Phosphorus)) were determined. The calibration period and validation period of the model were 2009–2010 and 2011–2012, respectively. In addition, water sampling was further conducted along the main stream of the Shuikou reservoir and the Youxi River to verify the coupling model and evaluate the effect of cage farming on water quality in the Shuikou reservoir and Youxi River by integrating with analysis scenarios on 27, February 2017 (Figure 3). Detailed information about the water quality is given in Table S1. The median error (ME) was employed to evaluate the goodness-of-fit of the EFDC model. The major purpose of model validation is to control the most results with little relative error [14].

(4) $$\mathrm{ME}=0.6745\sqrt{\frac{\sum_{i=1}^{n}{\left(\frac{Q_{obs}-Q_{cal}}{Q_{obs}}\right)}^{2}}{n-1}}$$

where *Q_obs_*, *Q_cal_* and *n* are the measured data, simulated data and the number of the data respectively.

### 2.5. Scenarios Development

Three scenarios were developed to evaluate the effect of climate variability and cage farming on water quality in the Minjinag river–reservoir system. Scenario 1 was designed to evaluate the sensitivity of water quality (represented by DO) in the Shuikou reservoir with respect to seasonal variation of the hydrologic condition and to pollutant export from tributaries.

Based on the weather condition of the Minjiang River watershed, three typical water seasons were listed as below (Table 1).

Based on the random sampling of the HSPF model, we proposed different classifications of precipitation, temperature (Table 2 and Table 3) and soil condition as the boundary conditions of HSPF model. In this regard, 72 typical scenarios of the Minjiang River watershed were proposed.

Scenario 2 was designed to evaluate the effects of pollutant export from the nearest tributary (i.e., Gutian River) (Ningde, China) on water quality (represented by NH_3_-N) in the Shuikou reservoir. According to the National Environmental Quality Standards for Surface Water (GB3838-2002), the water in the Gutian reservoir was not suitable for residential, industrial and agricultural uses (Figure S6). The average concentration of the NH_3_-N was 2.28 mg/L, and reached a maximum concentration of 8.24 mg/L. In this regard, different gradients of the initial NH_3_-N in a range of 2.28–8.24 mg/L were set in this scenario.

Scenario 3 was designed to evaluate the effect of farming cages in the Youxi River on water quality in the Shuikou reservoir (Figure S1). The inflow and outflow water quality of the farming cages was sampled as the initiation boundary of this scenario. The cage farming may become an additional source of the nutrient that can reduce the dissolved oxygen in this area, therefore the effect of cage farming in the Shuikou reservoir was evaluated by comparing the level of the DO, NH_3_-N and TP before and after the removal of farming cages from Youxi River. The water quality of farming cages was sampled as the boundary condition of the model in this study.

## 3. Results

### 3.1. Calibration and Validation Results

**Figure 4:**
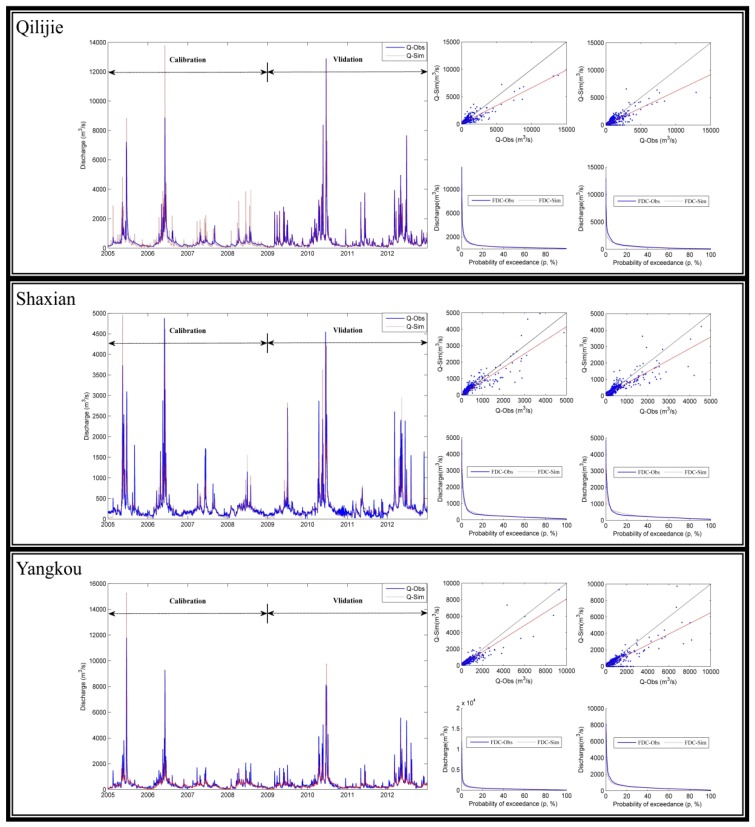
Daily flow calibration and validation for the HSPF model.

The calibration and validation results are shown in Figure 4. The simulated discharge fitted well with the observed values, with NSE and R^2^ over 0.7 (Table 4).

The results show that the RE for the water quality variables during the calibration period and validation period of HSPF model were 0.32–0.50 and 0.30–0.51, respectively (Table 5).

As for the EFDC model, Table 6 shows that the ME for the water quality simulation during the calibration period and validation period were 0.001–0.705 and 0.003–0.388, respectively. The ranges of the ME were within the ranges reported in other similar studies [14]. The coupled model was further verified by a recent investigation (Figure S7). In general, the coupled model can capture the trend of water quality dynamic in the river–reservoir system.

### 3.2. Seasonal Variation of Water Quality (Scenario 1)

**Figure 5:**
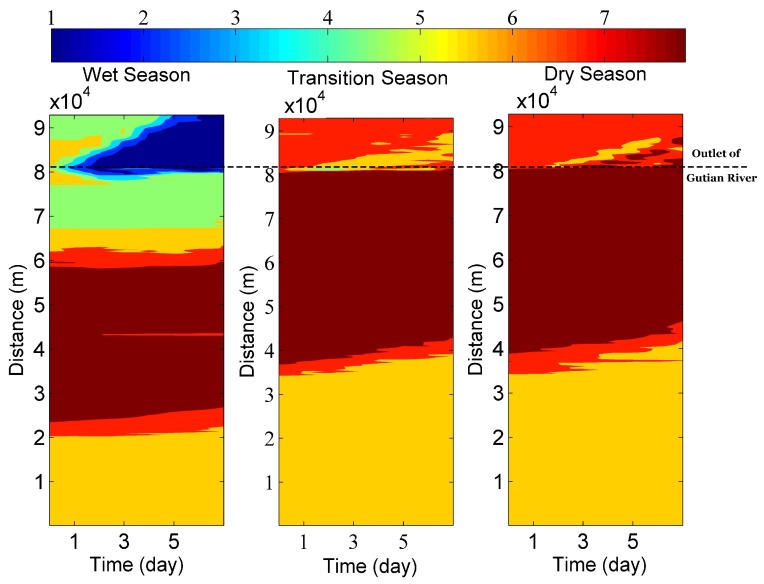
Simulation of DO across the Shuikou reservoir in three seasons. Note: The distance means the distance from the boundary of the upstream of the Shuikou reservoir.

The minimum DO in Shuikou reservoir was observed in the wet season, while the maximum was obtained in the dry season (Figure 5). The increasing runoff associated with increasing precipitation in the wet season from the Gutian River contributed greatly to water quality degradation in the outlet of the Shuikou reservoir. Compared to the Gutian River, the pollution loads from the Youxi River was not sensitive to the seasonal variation (Figure 5).

### 3.3. Sensitivity to Pollutant Export from the Gutian River (Scenario 2)

**Figure 6:**
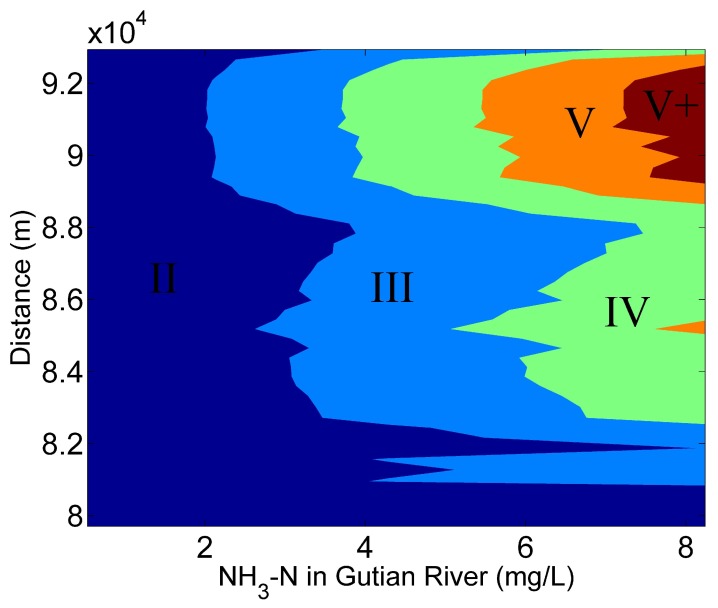
Simulation of NH_3_-N export from the Gutian River to the Shuikou reservoir.

The water quality in the downstream was more sensitive to the water quality degradation in the Gutian river. It was determined that the concentration of the NH_3_-N in the Gutian river should not be higher than 2.14 mg/L if downstream water is used for human consumption (Figure 6).

### 3.4. Effect of the Farming Cage (Scenario 3)

**Figure 7:**
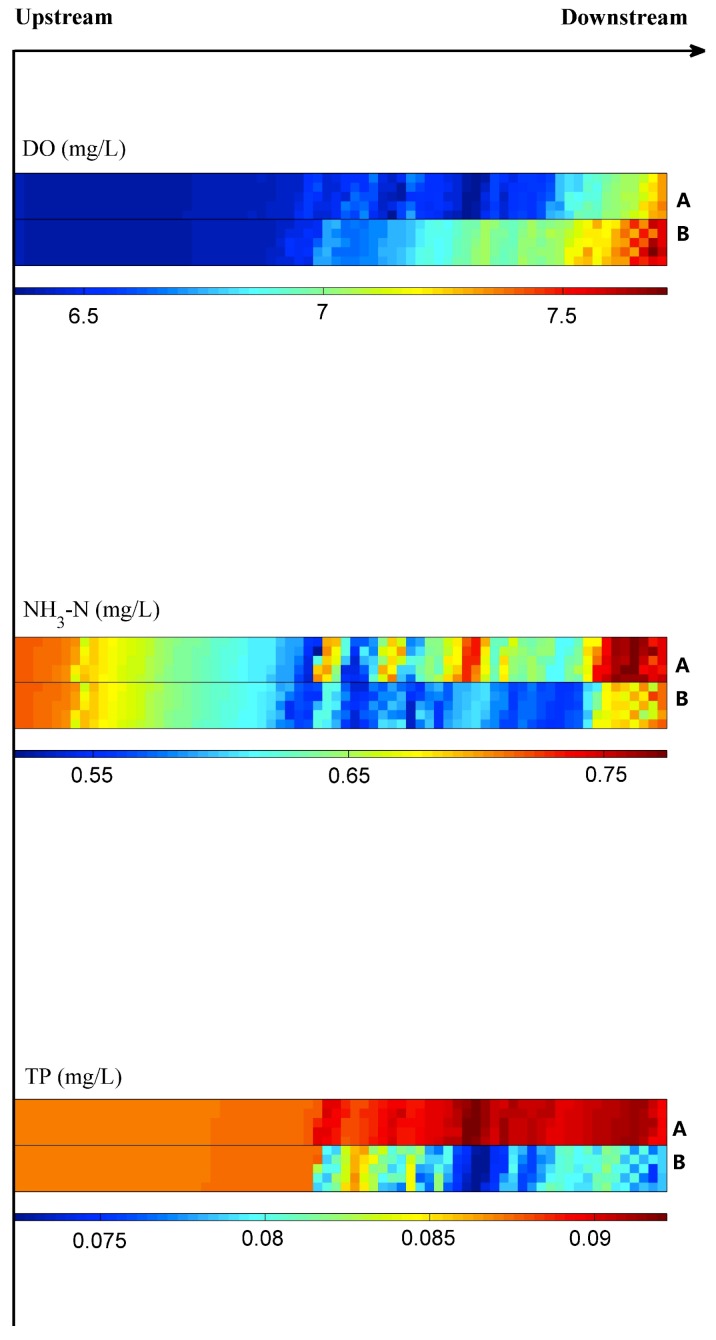
Simulation of DO, NH_3_-N and TP dynamics before **(A)** and after **(B)** removing farming cages.

**Figure 8:**
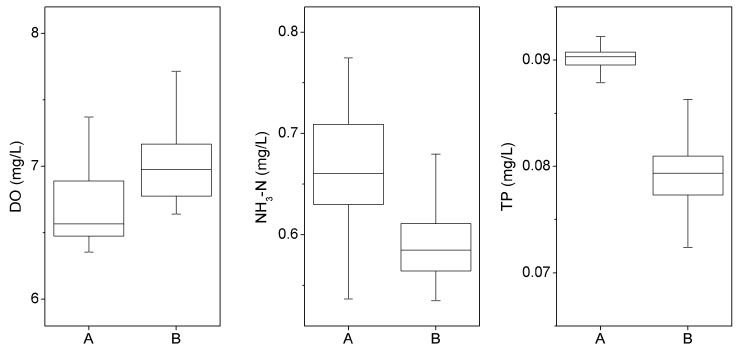
Statistics of simulation regarding DO, NH_3_-N and TP before **(A)** and after **(B)** removing cages in Shuikou Reservoir (based on the sampling sites 1–3, 7, 10–11).

The distribution of the DO, NH_3_-N and TP are presented in Figure 7. The water quality in the Suikou reservoir improved significantly after farming cages were removed. The DO in the downstream increased by 3%–10%, the concentration of NH_3_-N decreased by 5%–17% and the concentration of TP decreased by 6%–21% (Figure 8). However, the concentration of TP was still at a high level in the upstream of the Shuikou reservoir, which might influence the downstream water quality.

## 4. Discussions

### 4.1. Effectiveness of the Coupled Model

A coupled model can be an effective tool to understand a nutrient’s role and effects in hydrodynamic and ecosystem processes [11,12]. In this study we developed a coupled modeling approach that consisted of a watershed model (HSPF) and a hydrodynamic model (EFDC). To have a better understanding of the key factors influencing water quality in the Shuikou reservoir, the major sources of the pollutant was identified in this study with the coupled model.

The climate scenarios were set through the HSPF model, the pollutants from four major sub-watersheds along the Shuikou reservoirs as the initial boundary of EFDC model. In addition, the different streamflow regime of the tributary of the Shuikou reservoir and the transportation of the nutrient were also simulated by the EFDC model. The simulated values matched well with the observed values, and this coupled model was suitable for simulating processes in the Shuikou reservoir.

Recently, many researchers tried to couple the EFDC model with other watershed models in order to identify the effects of nonpoint source pollution [14,28,29]. In addition to that, in this study we also considered the pollutants derived from anthropogenic activities such as cage farming and damming in the tributary with the coupled model.

### 4.2. Scenarios Analysis for Delineating Nutrient Rate in the River-Reservoir System

Lakes and reservoirs serve as vital sources of drinking water and support valuable ecosystems across the globe [30]. The Gutian reservoir in the Minjiang River watershed has played an important role in providing drinking water for the local residents. Most of the concentration of DO in the Gutian River was lower than those found in the Youxi River in different seasons. The construction of reservoir in the Gutian River may lower the water movement in this area through rising the water level. The transport process characterized by water movement can change the positions of pollution load via advection and diffusion. Thus, the transport process may change the surrounding environments in the riverine system. It is reported that the changed water movement, which is induced by reservoirs or dam construction will alter the capacity of water exchange and pollutants dilution [31].

To further investigate the water quality in the outlet of the Shuikou reservoir, two strategies applied on the tributaries were evaluated: (1) to reduce the export of NH_3_-N in the Gutian River and (2) to remove the farming cages in the Youxi River. It was found that excessive phosphorus still discharges into the Shuikou reservoir after moving the farming cages in the Youxi River and it may influence the water quality of the Shuikou reservoir in the future. This similar result was also found in other large watersheds in China [3].

### 4.3. Management Implications

The findings of our study could be helpful for understanding potential source of the pollutants in the river–reservoir system. The nonpoint source pollution in the watershed might increase in the wet season [29,32]. Moreover, the reservoir construction tributary might amplify the export of the pollution in the wet season. Though the pollutant loads decreased obviously after the farming cages were moved, the water quality of the mainstream of the Shuikou reservoir might not stop degrading due to the excessive phosphorus discharge from the watershed. Some studies also indicated that the nonpoint source pollution might make a great contribution to water quality degradation for the large watershed [3]. Thus, in addition to the control of pollution loads in the river, local government might try induce the nonpoint source pollution in large watershed like the Minjiang River.

### 4.4. Limitation and Next Agenda

Our study concluded that the water movement might influence the pattern of nutrient export in the watershed (Figure 5). However, we need more quantitative information regarding water exchange and transport processes, so as to manage water quality effectively in the river–reservoir system. Recent studies show that water age, which is related to the water parcel transport time, can be used to examine the relationship between water age and water quality [3,33]. In the next agenda, we may design sound sampling strategy to link water exchange ability with water quality variation from the perspective of water age in the river–reservoir continuum.

## 5. Conclusions

A coupled model can be an effective tool to characterize the nutrient’s role and effects in the hydrodynamic and ecosystem processes. A coupled modeling approach was applied on a typical river–reservoir system within a large watershed in southeast China to investigate the main causes and key factors that influence the water quality. The results of calibration and validation of the models displayed a good agreement with field observations. This approach successfully reproduced the special characteristics of water quality in the Shuikou reservoir area. The proposed coupled modeling approach may effectively characterize waterway risks for water management. It was found that the reservoir may contribute greatly to water quality degradation as the result of decreasing water movement. The water quality may have been improved after farming cages were removed in the Youxi river. The findings of our study could be helpful for understanding potential sources of the pollutants in the river–reservoir system. The pattern of nonpoint source pollution might be influenced by the water season variation and reservoir construction. In the next agenda, we may design sound sampling strategy to link water exchange ability with water quality variation from the perspective of water age in the river–reservoir continuum.

## Figures and Tables

**Table 1:** Development of different water seasons.

Water Season	Period	Precipitation	Soil Condition	Temperature
Wet Season	Growth	V	III	IV
Transition Season	Growth	II	II	III
Dry Season	Dormancy	I	I	III

**Table 2:** Classifications of precipitation and temperature.

Classification	Precipitation (mm)	Temperature (°C)
I	0	5–15
II	0.1–9.9	15–0
III	10–24.9	20–25
IV	25–49.9	25–35
V	50–99.9	
VI	>100	

**Table 3:** Classifications of soil condition.

Classification	Precipitation Within 5 Days (mm)	
	Growth Period (March to November)	Dormancy Period (December to February)
I	<13	<36
II	13–28	36–53
III	>28	>53

**Table 4:** Performance of the HSPF model in streamflow simulation.

Station	Calibration		Validation	
	NSE (Nash–Sutcliffe Coefficient of Efficiency)	R^2^	NSE	R^2^
Shaxian	0.784	0.787	0.745	0.751
Yangkou	0.774	0.779	0.782	0.809
Qilijie	0.782	0.817	0.737	0.772

**Table 5:** Water quality calibration and validation in the HSPF model.

Stations	NH_3_-N		TP (Total Phosphorus)	
	Calibration	Validation	Calibration	Validation
	RE (Relative Error)	RE	RE	RE
Shaxian	0.50	0.51	0.32	0.30
Yangkou	0.34	0.42	0.50	0.33
Qilijie	0.32	0.54	0.49	0.30

**Table 6:** Water quality calibration and validation in the EFDC model.

Stations	DO (Dissolved Oxygen)		NH_3_-N		TP	
	Calibration	Validation	Calibration	Validation	Calibration	Validation
	ME (Median Error)	ME	ME	ME	ME	ME
Youxi	0.001	0.027	0.003	0.0232	0.003	0.008
Taxia	0.012	0.001	0.072	0.002	0.080	0.001
Zhanghuban	0.243	0.143	0.705	0.298	0.438	0.326
Shuikou	0.331	0.266	0.327	0.388	0.295	0.296

## Supplementary Materials

The following are available online at https://www.mdpi.com/1660-4601/16/16/2949/s1, Figure S1: Farming cages of Shuikou reservoir in 2016 **(A)** and the dead fish event of Shuikou reservoir in 2011 **(B)**, Figure S2: Water quality of Shuikou reservoir (2000–2012), Figure S3: Land use of Minjiang watershed (2014), Figure S4: Soil classification of Minjiang River watershed, Figure S5: Location of weather station, Figure S6: Trend of NH_3_-N of the Gutian River, Figure S7: Validation of DO, NH_3_ and TP using the sampling data in February 2017, Table S1: Water sampling on 27, February 2017.
