# Supplementary material for: A Coupled Modeling Approach for Water Management in a River–Reservoir System
Source: Int J Environ Res Public Health. 2019 Aug 16;16(16):2949. doi: 10.3390/ijerph16162949 (PMC6719991; doi:10.3390/ijerph16162949)
Supplement: Supplementary file 1 [file ijerph-16-02949-s001.pdf]

An Coupled Modeling Approach for Water Management in a River-reservoir System

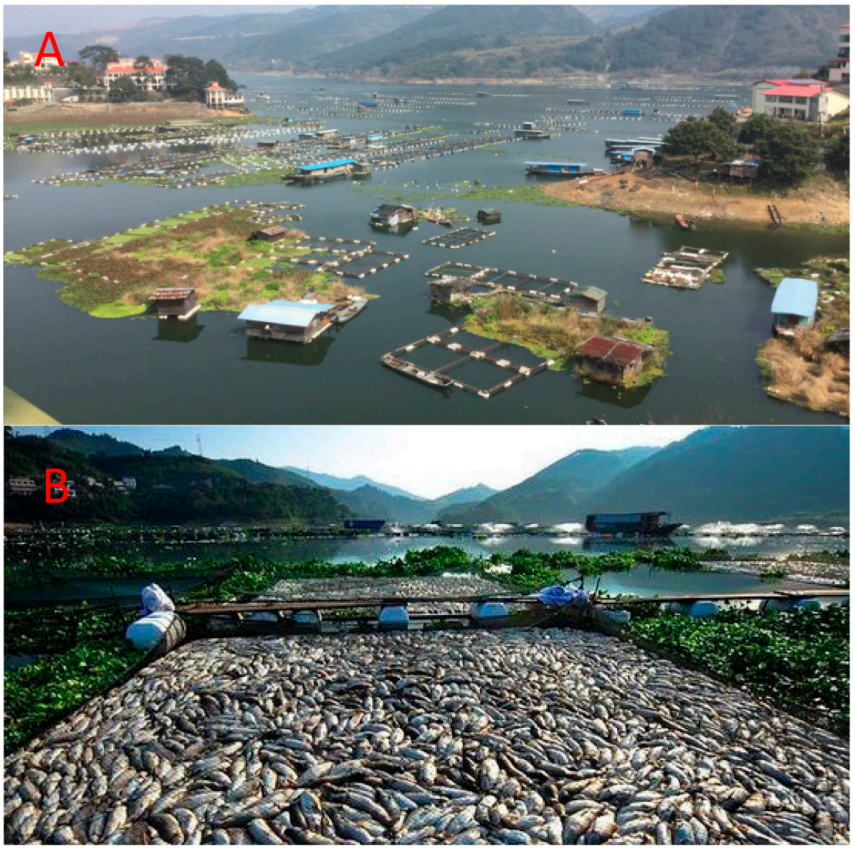

Figure S1. Farming Cages of Shuikou Reservior in 2016 (**A**) and the Dead Fish Event of Shuikou Reservior in 2011 (**B**).

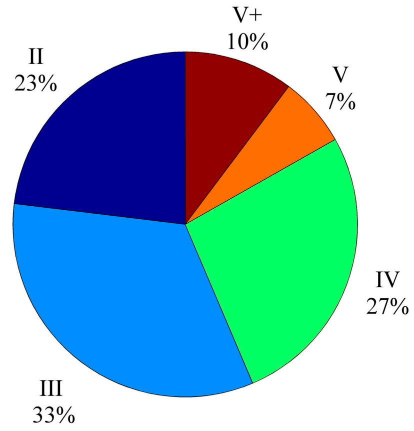

Figure S2. Water quality of Shuikou reservoir (2000–2012).

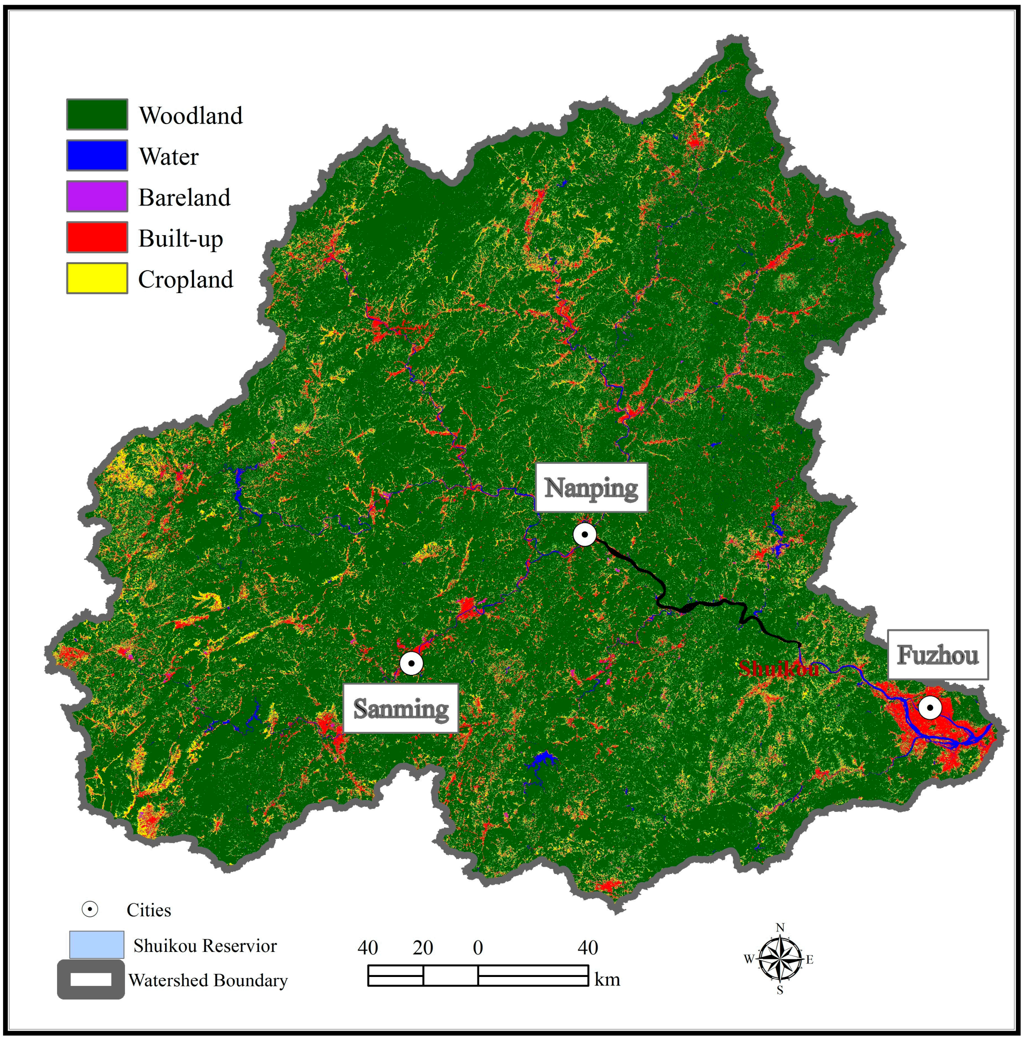

Figure S3. Land use of Minjiang Watershed (2014).

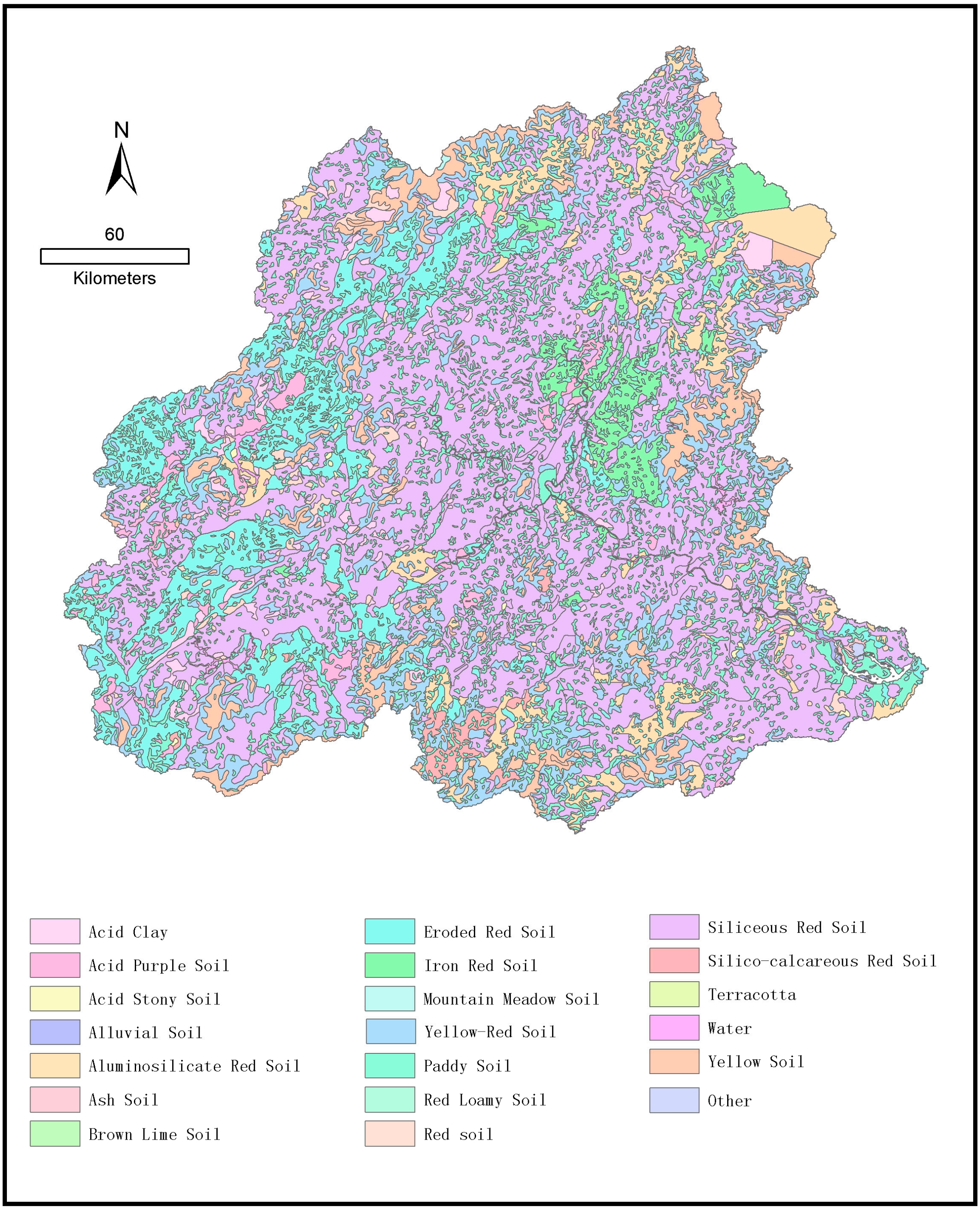

Figure S4. Soil classification of Minjiang River Watershed.

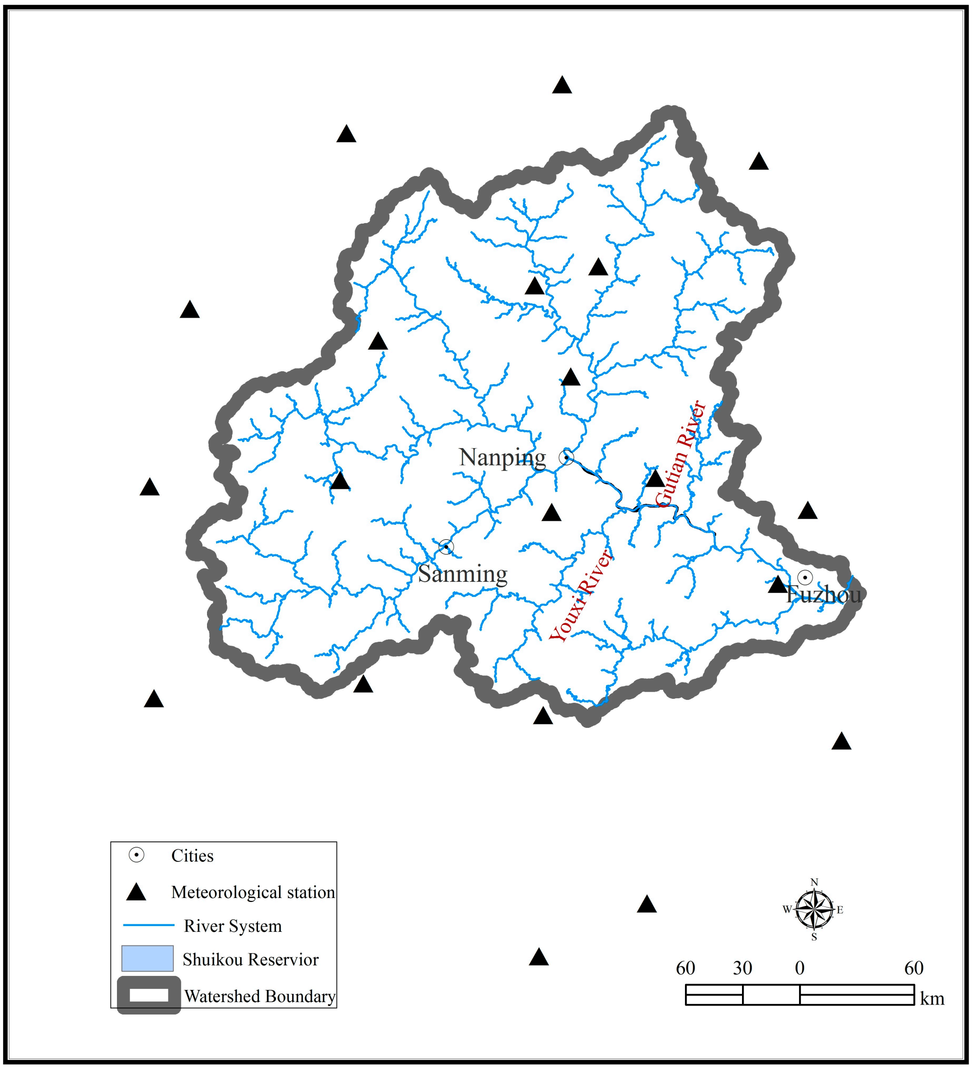

Figure S5. Location of weather station.

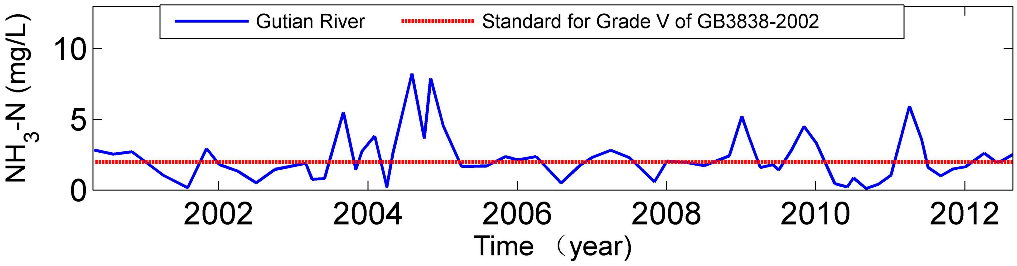

Figure S6. Trend of NH_3-N of the Gutian River.

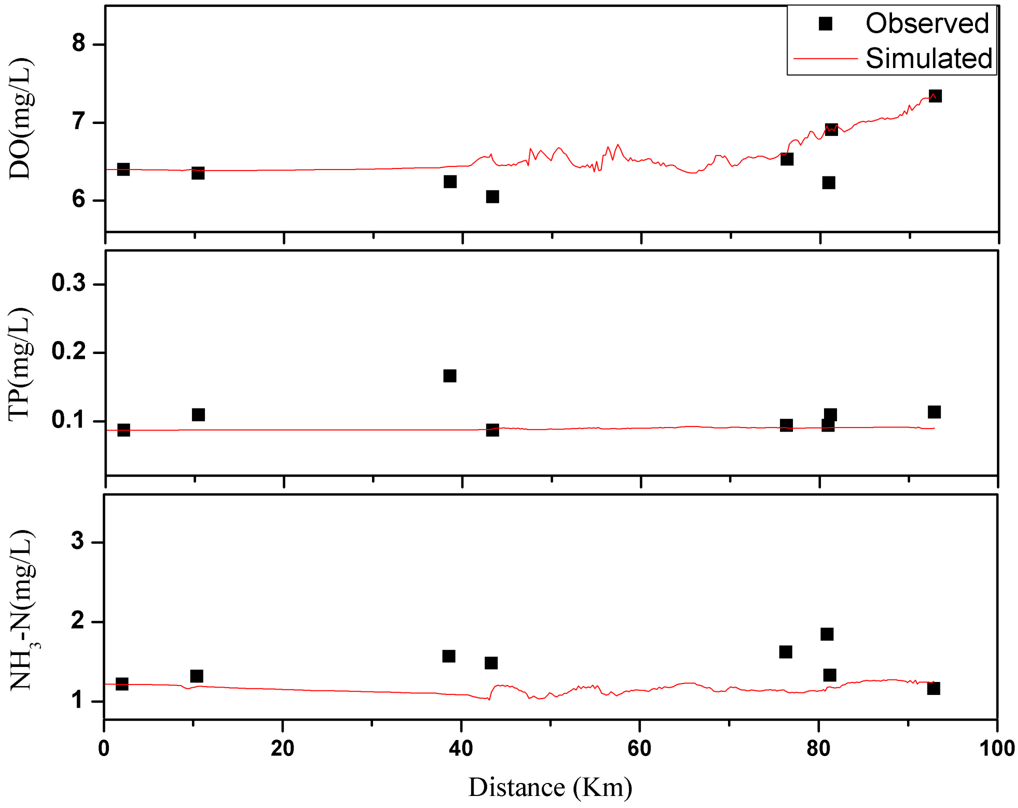

Figure S7. Validation of DO, NH_3, TP using the sampling data on February 2017.

Table S1. Water sampling on 27 February 2017.

Sampling Site	Latitude	Longitude	DO(mg/L)	NH_3-N(mg/L)	TP(mg/L)
1	26°37'57.4''	118°11'54.6''	6.4	0.719	0.087
2	26°35'06.2''	118°15'37.1''	6.35	0.816	0.109
3	26°25'18.0''	118°24'19.1''	6.24	1.067	0.166
4	26°17'32.4''	118°20'33.3''	7.12	0.747	0.064
5	26°21'47.2''	118°23'20.3''	6.28	0.983	0.094
6	26°23'44.4''	118°26'59.0''	6.05	0.76	0.087
7	26°21'02.5''	118°41'04.3''	6.53	1.123	0.094
8	26°25'54.1''	118°45'32.3''	8.07	1.485	0.109
9	26°22'31.2''	118°43'44.8''	6.23	1.345	0.094
10	26°22'16.2''	118°43'41.0''	6.91	0.83	0.109
11	26°17'45.3''	118°49'32.3''	7.34	0.663	0.113
